# Risk and early predictive factors of anastomotic leakage in laparoscopic low anterior resection for rectal cancer

**DOI:** 10.1186/s12957-019-1716-3

**Published:** 2019-11-02

**Authors:** Masahiro Fukada, Nobuhisa Matsuhashi, Takao Takahashi, Hisashi Imai, Yoshihiro Tanaka, Kazuya Yamaguchi, Kazuhiro Yoshida

**Affiliations:** Department of Surgical Oncology, Graduate School of Medicine, Gifu University, 1-1 Yanagido, Gifu City, Gifu 501-1194 Japan

**Keywords:** Rectal cancer, Anastomotic leakage, Double-stapling technique, Laparoscopic low anterior resection

## Abstract

**Background:**

In recent years, laparoscopic surgery has been widely used for rectal cancer. In laparoscopic rectal surgery, a double-stapling technique (DST) anastomosis using a stapling device is considered a relatively difficult procedure. Postoperative anastomotic leakage (AL) is a major complication related to patients’ quality of life and prognosis.

**Methods:**

This study was a retrospective, single-institution study of 101 rectal cancer patients who underwent laparoscopic low anterior resection (LAR) with DST anastomosis (excluding simultaneous resection of other organs and construction of protective diverting stoma) between February 2008 and November 2017 at the Gifu University Graduate School of Medicine. This study aimed to identify risk and early predictive factors of AL.

**Results:**

Among 101 patients, symptomatic AL occurred in 13 patients (12.9%), of whom 10 were male and 3 were female. Their median BMI was 22.7 kg/m^2^ (range, 17.9–26.4 kg/m^2^).

Among the pre- and intraoperative factors, AL was significantly associated with tumor location (lower rectum), distance from the anal verge (< 6 cm), intraoperative blood loss (≥ 50 ml), and the number of linear staples (≥ 2) in univariate analysis. In multivariate analysis, only intraoperative blood loss (≥ 50 ml, odds ratio [OR] 4.59; 95% confidence interval [CI] 1.04–19.52; *p* = 0.045) was identified as an independent risk factor for AL.

Among the postoperative factors, AL was significantly associated with tachycardia-POD1 (≥ 100 bpm), CRP-POD3 (≥ 15 mg/dl), fever on postoperative day (fever-POD) 3 (≥ 38 °C), and first defecation day after surgery (< POD3) in univariate analysis. In multivariate analysis, fever-POD3 (≥ 38 °C, OR 30.97; 95% CI 4.68–311.22; *p* = 0.0003) and first defecation day after surgery (< POD3, OR 5.82; 95% CI 1.34–31.30; *p* = 0.019) were identified as early predictive factors for AL.

**Conclusion:**

In this study, intraoperative blood loss was an indicator of difficulty in a transection and anastomosing procedure, and fever-POD3 and early first defecation day after surgery were independent early predictive factors for AL. Careful surgery using an appropriate technique and standardized procedures with minimal bleeding and careful postoperative management paying attention to fever and defecation may prevent the onset and severity of AL.

## Introduction

With the development of laparoscopic surgery, laparoscopic rectal surgery has become a widespread intervention for rectal cancer; in fact, according to a report by the Japanese Society for Endoscopic Surgery (JSES), there have been 10,288 cases in 2017 alone. Laparoscopic surgery is now the standard operation instead of open surgery for rectal cancer.

Postoperative anastomotic leakage (AL) is a major complication in laparoscopic rectal surgery that is related to patients’ quality of life (QOL) and prognosis, especially morbidity, mortality, functional defects, and oncologic outcomes [1–3]. Despite technical improvements and instrumental developments, rectal transection and double-stapling technique (DST) anastomosis using linear and circular staples are relatively difficult. Hence, the AL rate remains at 6.3–13.7% [4–9].

In addition, when AL occurs, re-operation and treatment for peritonitis are required. Hence, postoperative hospital stays become longer. In the case of advanced cancer with lymph node metastasis, the introduction of postoperative adjuvant chemotherapy may be delayed, which may lead to an increased recurrence rate and poor prognosis.

The present study had two clinical objectives: (1) identifying risk factors by the evaluation of pre- and intraoperative factors and (2) identifying early predictive factors by the evaluation of postoperative factors. Our aim is to improve patient outcomes by identifying these factors to prevent the occurrence and severity of AL.

## Materials and methods

### Study population

**Fig. 1:**
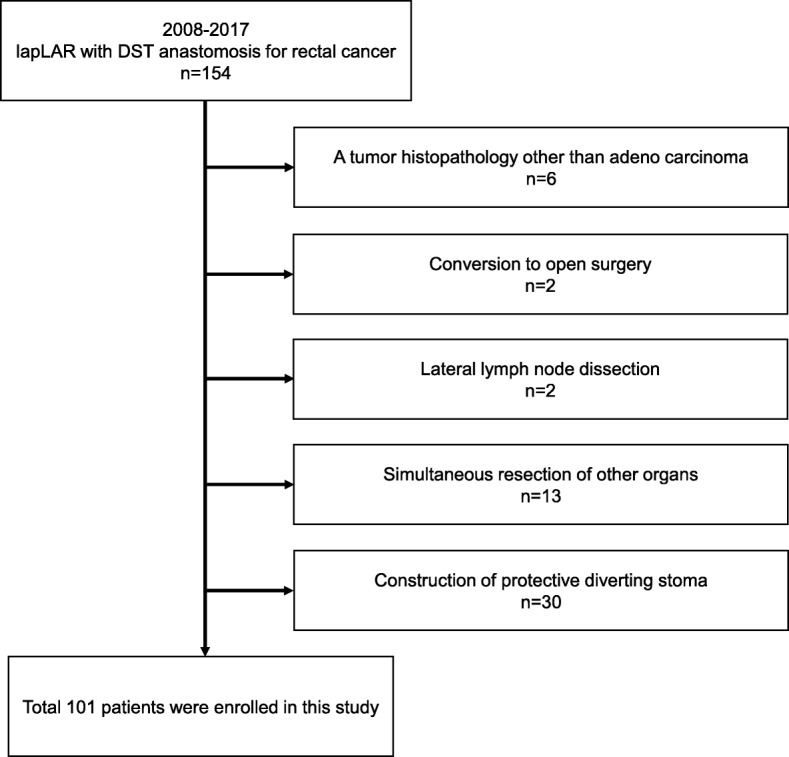
Exclusion criteria

A total of 154 patients consecutively underwent elective laparoscopic low anterior resection (LAR) with a DST anastomosis at the Department of Surgical Oncology, Gifu University Graduate School of Medicine, between February 2008 and November 2017. Among those patients, 53 were excluded for tumor histopathology other than adenocarcinoma (*n* = 6); conversion to open surgery (*n* = 2); lateral lymph node dissection (*n* = 2); simultaneous resection of other organs (*n* = 13); and construction of protective diverting stoma (DS) (*n* = 30) (Fig. 1). Ultimately, a total of 101 patients with primary rectal cancers were included in this study. We included only symptomatic AL requiring therapeutic interventions or an operation in the present study. Therefore, DS cases were excluded. No patients had preoperative chemotherapy or chemoradiotherapy. In our department, primary resection is routinely performed before chemotherapy, even for the cases with distant metastases. In addition, preoperative chemotherapy or chemoradiotherapy is selected for difficult cases such as bulky tumors or extramural invasion. Preoperative treatment should be performed in those cases after construction of the colostomy for safer treatment.

The location of the tumor and distance from the anal verge was determined by computed tomography (CT), colonoscopy (CS), and either CT colonography (CTC) or barium enema (BE) preoperatively and confirmed during surgery. The maximum tumor diameter, clinical tumor depth, and lymph node metastasis were also determined by CT, CS, and CTC or BE preoperatively and confirmed through postoperative histopathological examination findings. Clinically positive lymph node metastasis was defined as nodes with a short-axis diameter of at least 8 mm by CT. In the present study, postoperative fever was defined as a body temperature ≥ 38 °C and postoperative tachycardia as a heart rate ≥100 bpm.

Patient-, tumor-, and surgery-related variables were included in the analysis. The patient-related variables were age, sex, body mass index (BMI), American Society of Anesthesiologists physical status classification (ASA), diabetes mellitus, serum albumin (Alb), and prognostic nutritional index (PNI). The tumor-related variables were tumor location, maximum tumor diameter, distance from the anal verge, circumferential tumor, preoperative stent placement, and clinical and pathological Union for International Cancer Control-TNM classification (8th edition) [10]. The intraoperative surgery-related variables were operation time, blood loss, lymph node dissection level (D2 or D3), left colic artery (LCA) preservation, mobilization of the splenic flexure, number of linear staples used for rectal transection, size of the circular staple, placement of the transanal drain, and surgeon qualifications. The postoperative surgery-related variables were white blood cell (WBC) count, C-reactive protein (CRP) levels, fever, tachycardia, watery stools after surgery, and first defecation day after surgery. To resolve clinical questions, they were classified into pre-, intra-, and postoperative factors.

### Surgical methods

All procedures were conducted at our department by an experienced laparoscopic colorectal surgeon qualified by the Endoscopic Surgical Skill Qualification System of the JSES. “Qualified surgeon,” one of the surgery-related factors, indicates a case in which a qualified surgeon completed the operation as the main operator. In all other cases, a qualified surgeon supervised as the first assistant.

According to the recommended clinical pathway, all patients received standard bowel preparation by the administration of magnesium citrate and sennoside 2 days before surgery (only mechanical prophylaxis, no antibiotic prophylaxis). In cases where a stent was placed, bowel preparation was slowly performed for 2–3 weeks after placement. Thereafter, a total colonoscopy was performed to confirm that there were no other lesions and that sufficient preparation was done before the surgery was approved and carried out.

We routinely performed high ligation of the inferior mesenteric artery, although preservation of the LCA was considered depending on blood vessel condition. The splenic flexure was mobilized either totally or partially, depending on the bowel length. After mobilization of the descending colon, tumor-specific mesorectal dissection was performed by sharp mesorectal dissection with a nerve-preserving technique. After clamping the distal side of the tumor to wash out the rectum, the rectum was transected using a linear staple (Echelon 60 or Powered Echelon 60, Gold cartridge, Ethicon Endo-Surgery, Cincinnati, OH, USA). After the surgical specimen was removed through the small incision, the anvil head of a circular staple was positioned in the proximal colon. The circular staple (CDH, 25 mm or 29 mm, Ethicon) was inserted through the rectum, and DST anastomosis was completed intracorporeally. Airtightness was routinely tested by transanal instillation of air. If the leak test was positive, an intracorporeal reinforcement suture with 3–0 vicryl was placed at the weak point. The placement of a pelvic drain was routinely performed, and placement of the transanal drain (pleated drain-soft type, 10 mm, Sumitomo Bakelite Company Limited, Shinagawa, Tokyo, Japan) was performed depending on the location of the tumor and the height of the anastomosis from the anal verge. The transanal drain was removed 4–5 days after surgery.

### Definition of anastomotic leakage

AL was investigated in the presence of clinical leakage signs such as the discharge of pus or feces from the pelvic drain and evidence of peritonitis, including abdominal pain, tenderness, fever, tachycardia, or severe inflammation in blood tests. If leakage was suspected, CT was performed to check for AL. Diagnosis required positive findings on CT, such as an abscess and fluid collection or air bubbles surrounding the anastomosis site. Asymptomatic anastomosis leakage was not considered as contrast enemas were not routinely performed in our department.

### Statistical analysis

Continuous variables were expressed as median values, while categorical and ordinal variables were expressed as frequencies (percentages). For comparisons of variables between the non-AL and AL groups, Fisher’s exact test was used for categorical variables, and Wilcoxon rank sum tests were used for continuous and ordinal variables. To test the independence of risk and early predictive factors for AL, significant variables in univariate analyses were included in a final model of logistic regression. All statistical analyses were performed using JMP software (SAS Institute Inc., Cary, NC, USA).

## Results

### Patient and tumor characteristics

In total, 101 consecutive patients underwent laparoscopic LAR with DST anastomosis, 53 (52.5%) were male and 48 (47.5%) were female. The median age was 64 years (range, 18–83 years) and their median BMI was 22 kg/m^2^ (range, 15.4–29.7 kg/m^2^). Twenty-five patients (24.7%) had rectosigmoid (RS) cancer, 63 (62.4%) had upper rectal (Ra) cancer, and the remaining 13 (12.9%) had lower rectal (Rb) cancer.

### Anastomotic leakage

Among the patients, symptomatic AL occurred in 13 patients (12.9%), of whom 10 were male and 3 were female. Their median BMI was 22.7 kg/m^2^ (range, 17.9–26.4 kg/m^2^). The AL rate was 12.0% (3/25) in patients with RS cancer, 7.9% (5/63) in Ra cancer, and 38.5% (5/13) in Rb cancer. AL requiring re-operation (stoma construction) occurred in 5 cases (38.5%), while there were 8 cases (61.5%) of AL that did not require re-operation and were treated by irrigation and drainage through the pelvic drain. Antibiotics were administered in all AL cases until the inflammatory response was reduced or there were no signs of inflammation. The median time at which AL was confirmed was POD 4 (range, 1–10 days), and the median time until hospital discharge was 43 days post-surgery (range, 24–242 days). There were no deaths related to AL in this study.

### Patient-related factors for AL

Patient-related factors are summarized in Table 1. No significant differences were found, which was consistent even when converting continuous and ordinal variables into categorical variables [age (≥ 75 years/< 75 years), BMI (≥ 25 kg/m^2^/< 25 kg/m^2^), ASA (≥ 2/< 2), Alb (≥ 3.5 g/dl/< 3.5 g/dl), and PNI (≥ 45/< 45)]. In this study, we set a BMI cutoff value of ≥ 25 kg/m^2^ based on the definition of obesity in the Japanese Society for the Study of Obesity (JASSO) instead of 30 kg/m^2^ as per the International Federation of Surgery for Obesity and Metabolic disorder (IFSO).

**Table 1:** Patients related factors for AL

Patient related factor	Leakage (−), *n* = 88	Leakage (+), *n* = 13	*p* value
Age (years), median [range]	64.5 [18~83]	60 [36~79]	0.39
Sex, *n* = No. (%)			
Male	43 (48.9)	10 (76.9)	0.077
Female	45 (51.1)	3 (23.1)	
Body mass index, median [range]	21.75 [15.4~29.7]	22.7 [17.9~26.4]	0.24
ASA, *n* = No. (%)			
1	29 (33.0)	5 (38.5)	0.82
2	58 (65.9)	8 (61.5)	
3	1 (1.1)	0 (0)	
Diabetes mellitus, *n* = No. (%)			
Yes	16 (18.2)	5 (38.5)	0.14
No	72 (81.8)	8 (61.5)	
Albumin (g/dl), median [range]	4.3 [2.7~4.9]	4.4 [3.2~4.9]	0.79
PNI, median [range]	52.1 [30.6~63.9]	53.4 [40.6~65.9]	0.50

*AL* anastomotic leakage, *ASA* American Society of Anesthesiologists physical status classification, *PNI* prognostic nutritional index = 10 × Alb(g/dl) + 0.005 × total lymphocyte count (mm^3^)

^✝^*p* < 0.05

### Tumor-related factors for AL

Clinical and pathological tumor-related factors are summarized in Table 2. AL was significantly associated with tumor location (*p* = 0.031) and distance from the anal verge (*p* = 0.040). There were significant differences in tumor location (Rb, *p* = 0.0046) and distance from the anal verge (< 6 cm, *p* = 0.0090) after converting continuous and ordinal variables into categorical variables [tumor location (Rb/not Rb), tumor diameter (≥ 40 mm/< 40 mm), distance from the anal verge (≥ 6 cm/< 6 cm), cT (≥ 3/< 3), cN (positive/negative), cStage (≥ III/< III), pT (≥ 3/< 3), pN (positive/negative), and fStage (≥ III/< III)].

**Table 2:** Tumor related factors for AL

Tumor related factors	Leakage (−), *n* = 88	Leakage (+), *n* = 13	*p* value
Clinical			
Tumor location, *n* = No. (%)			
Rs	22 (25.0)	3 (23.0)	0.031✝
Ra	58 (65.9)	5 (38.5)	
Rb	8 (9.1)	5 (38.5)	
Tumor diameter (mm), median [range]	35 [5~112]	42 [10~100]	0.36
Anal verge (cm), median [range]	13 [5~30]	10 [5~20]	0.040✝
Circumferential tumor, *n* = No. (%)			
Yes	19 (21.8)	4 (30.8)	0.49
No	68 (78.2)	9 (69.2)	
Stent placement, *n* = No. (%)			
Yes	5 (5.7)	2 (15.4)	0.22
No	83 (94.3)	11 (84.6)	
cT, *n* = No. (%)			
1	18 (20.4)	3 (23.1)	0.94
2	18 (20.4)	2 (15.4)	
3	35 (39.8)	6 (46.1)	
4	17 (19.4)	2 (15.4)	
cN, *n* = No. (%)			
0	60 (68.2)	7 (54.0)	0.49
1	18 (20.5)	3 (23.0)	
2	10 (11.3)	3 (23.0)	
cStage, *n* = No. (%)			
I	36 (40.9)	5 (38.5)	0.83
II	21 (23.9)	2 (15.4)	
III	23 (26.1)	4 (30.7)	
IV	8 (9.1)	2 (15.4)	
Pathological			
pT, *n* = No. (%)			
1	24 (27.3)	3 (23.1)	0.74
2	19 (21.6)	4 (30.8)	
3	30 (34.1)	5 (38.4)	
4	15 (17.0)	1 (7.7)	
pN, *n* = No. (%)			
0	57 (64.8)	6 (46.1)	0.098
1	20 (22.7)	2 (15.4)	
2	11 (12.5)	5 (38.5)	
fStage, *n* = No. (%)			
I	37 (42.0)	5 (41.7)	0.15
II	17 (19.3)	0 (0.0)	
III	26 (29.6)	5 (41.7)	
IV	8 (9.1)	2 (16.6)	

*AL* anastomotic leakage

^✝^*p* < 0.05

### Surgery-related factors for AL

Surgery-related factors are summarized in Table 3. AL was significantly associated with the number of linear staples (*p* = 0.046), tachycardia-POD1 (*p* = 0.023), CRP-POD3 (*p* = 0.036), fever-POD3 (*p* < 0.0001), and first defecation day after surgery (*p* = 0.022). There were significant differences in intraoperative blood loss (≥ 50 ml, *p* = 0.012), CRP-POD 3 (≥ 15 mg/dl, *p* = 0.046), and first defecation day (< POD3, *p* = 0.0059) after converting continuous and ordinal variables into categorical variables [operation time (≥ 240 min/< 240 min), intraoperative blood loss (≥ 50 ml/< 50 ml), WBC-POD1 (≥ 10,000/μl/< 10,000/μl), CRP-POD1 (≥ 5 mg/dl/< 5 mg/dl), WBC-POD3 (≥ 10,000/μl/< 10,000/μl), CRP-POD3 (≥ 15 mg/dl/< 15 mg/dl), and first defecation day (≥ POD3/< POD3)].

**Table 3:** Surgery related factors for AL

Surgery related factors	Leakage (−), *n* = 88	Leakage (+), *n* = 13	*p* value
Intraoperative			
Operation time (min), median [range]	233 [151~438]	263 [157~376]	0.32
Blood loss (ml), median [range]	10 [0~180]	15 [0~1115]	0.15
Lymph node dissection, *n* = No. (%)			
D2	18 (20.5)	1 (7.7)	0.45
D3	70 (79.5)	12 (92.3)	
LCA preserving, *n* = No. (%)			
Yes	36 (40.9)	4 (30.8)	0.56
No	52 (59.1)	9 (69.2)	
Mobilization of splenic flexure, *n* = No. (%)			
Yes	3 (3.4)	1 (7.7)	0.43
No	85 (96.6)	12 (92.3)	
Number of linear staple, *n* = No. (%)			
1	66 (75.0)	6 (46.1)	0.046^✝^
2	22 (25.0)	7 (53.9)	
Size of circular staple (mm), *n* = No. (%)			
25	7 (8.1)	1 (7.7)	1.00
29	80 (91.9)	12 (92.3)	
Trans anal drain, *n* = No. (%)			
Yes	51 (58.0)	8 (61.5)	1.00
No	37 (42.0)	5 (38.5)	
Qualified surgeon, *n* = No. (%)			
Yes	65 (73.9)	11 (84.6)	0.51
No	23 (26.1)	2 (15.4)	
Postoperative			
WBC (/μl) median [range]			
POD1	8280 [2790~13,510]	8340 [5640~12,440]	0.069
POD3	6975 [3220~15,450]	8100 [3390~17,570]	0.15
CRP (mg/dl) median [range]			
POD1	4.95 [1.61~12.7]	4.43 [2.35~14.7]	0.75
POD3	6.86 [0.37~23.4]	8.77 [1.24~34.6]	0.036^✝^
Fever (≧ 38 °C), *n* = No. (%)			
POD1	40 (45.5)	9 (69.2)	0.14
POD3	5 (5.7)	7 (53.8)	< 0.0001^✝^
Tachycardia (≧ 100 bpm), *n* = No. (%)			
POD1	6 (6.8)	4 (30.8)	0.023^✝^
POD3	7 (8.0)	1 (8.0)	1.00
Watery stool, *n* = No. (%)			
Yes	34 [39.0]	6 [50.0]	0.54
No	53 [61.0]	6 [50.0]	
First defecation day (POD), median [range]	4 [1~13]	2 [0~7]	0.022^✝^

*AL* anastomotic leakage, *LCA* left colic artery, *WBC* white blood cell, *CRP* C-reactive protein, *POD* postoperative day

^✝^*p* < 0.05

### Pre-, intra-, and postoperative factors

**Fig. 2:**
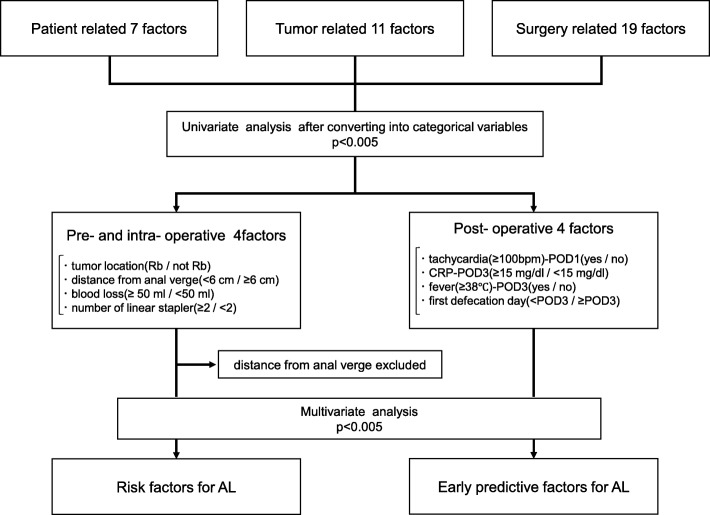
Analysis flow chart for identifying risk and early predictive factors for anastomotic leakage

To resolve clinical questions, we classified patient-, tumor-, and surgery-related categorical factors, which were significantly different in univariate analysis, into pre-, intra-, and postoperative factors (Fig. 2).

Among the pre- and intraoperative factors, AL was significantly associated with the following four factors in univariate analysis: tumor location (Rb), distance from the anal verge (< 6 cm), intraoperative blood loss (≥ 50 ml), and the number of linear staple (≥ 2). In multivariate analysis, distance from the anal verge (< 6 cm) was excluded to avoid confounding with tumor location (Rb); only intraoperative blood loss (≥ 50 ml) maintained a significant correlation with AL (Table 4; odds ratio (OR) 4.59; 95% confidence interval (CI) 1.04–19.52; *p* = 0.045).

**Table 4:** Multivariate analysis of pre- and intraoperative factors for AL

Pre-and intraoperative factors	Odds ratio	95% CI	*p* value
Tumor location (Rb/not Rb)	2.69	[0.54~11.68]	0.21
Blood loss (≥ 50 ml/< 50 ml)	4.59	[1.04~19.52]	0.045^✝^
Number of linear staple (≥ 2/< 2)	2.34	[0.63~8.61]	0.20

*AL* anastomotic leakage

^✝^*p* < 0.05

Among the postoperative factors, AL was significantly associated with the following four factors in univariate analysis: tachycardia-POD1 (≥ 100 bpm), CRP-POD3 (≥ 15 mg/dl), fever-POD3 (≥ 38 °C), and first defecation day after surgery (< POD3). In multivariate analysis, fever-POD3 (≥ 38 °C) and first defecation day after surgery (< POD3) remained significantly correlated with AL (Table 5; OR 30.97; 95% CI 4.68–311.22; *p* = 0.0003 and OR 5.82; CI 1.34–31.30; *p* = 0.019, respectively).

**Table 5:** Multivariate analysis of postoperative factors for AL

Postoperative factors	Odds ratio	95% CI	*p* value
Tachycardia (≥ 100 bpm)-POD1 (yes/no)	4.84	[0.58~34.59]	0.14
CRP-POD3 (≥ 15 mg/dl/< 15 mg/dl)	0.28	[0.002~2.51]	0.27
Fever (≥ 38 °C)-POD3 (yes/no)	30.97	[4.68~311.22]	0.0003^✝^
First defecation day (< POD3/≥ POD3)	5.82	[1.34~31.30]	0.019^✝^

*AL* anastomotic leakage, *CRP* C-reactive protein, *POD* postoperative day

^✝^*p* < 0.05

## Discussion

AL is a major complication of laparoscopic rectal surgery. It is associated with postoperative morbidity, mortality, functional defects, and oncologic outcomes [1–3]. Several risk factors have been reported for AL after open LAR [11–15]. Recently, some studies have also examined risk factors for AL after laparoscopic LAR [4, 16–26]. The devices and techniques used for laparoscopic LAR are different from open LAR, suggesting that the risk factors for AL may also differ between laparoscopic and open LAR. According to their studies, anastomotic level, number of linear staples, sex, BMI, smoking, alcohol intake, previous abdominal surgery, preoperative CRT, tumor location, stage, operation time, blood loss, transfusion, and pre-compression before firing have been reported to be risk factors for AL after laparoscopic LAR. In the present study, analysis of preoperative and intraoperative factors suggested that intraoperative blood loss, distance from the anal verge, and number of linear staples may be candidates for risk factors.

Firstly, in some studies, intraoperative blood loss has been reported to be an independent risk factor for AL [21–23, 25, 26]. In the present study, there was no significant difference in blood loss as a continuous variable, but a significant difference was observed only when 50 ml was used as a cutoff value. This indicates that AL does not directly manifest due to bleeding, and intraoperative blood loss is likely to be a surrogate for the difficulty of the surgery. The results of this study suggest that intraoperative blood loss of more than 50 ml may be one of the objective indicators of a challenging transection and anastomosing procedure. Therefore, performing well-coordinated laparoscopic surgery using standardized procedures could help to reduce the intraoperative blood loss and to create a proper anastomosis.

Secondly, several studies have reported that tumor location and distance from the anal verge are risk factors for laparoscopic LAR [17–21, 24]. Choi et al. [19] reported that the AL rate was 10 times higher (20.6% vs. 2.3%) when the anastomotic region was located within 5 cm of the anal verge in a series of 156 patients undergoing laparoscopic LAR without DS. It is hypothesized that tumor location and distance from the anal verge can reflect technical difficulties and affect anastomotic tension and blood supply. In the present study, there were no statistically significant differences between these factors in multivariate analysis; however, we considered that they are very likely to be risk factors for AL. In our department, DS construction has been performed routinely in cases of Rb cancer requiring transection just above the anal canal (< 5 cm from the anal verge).

Thirdly, some previous studies reported that the number of linear staples used for rectal transection was a risk factor for AL [4, 17–20, 24]. There is a concern that an increased number of staple firings may lead to small defects between staple lines and cause AL. Furthermore Kim et al. [17] found that more than two staple firings was associated with AL, and the number of linear staples was significantly higher in males, patients with a tumor closer to the rectal verge, and in those with longer operation times. Therefore, the number of linear staples seems to be both a direct and indirect risk factor for AL. Although there was no statistical significance in multivariate analysis, laparoscopic surgeons need to refine their technique to transect the rectum using one linear staple when possible.

Although in univariate analysis, sex (male) showed a significant tendency (*p* = 0.077), no other factors showed significant differences. The nutritional index in this test was analyzed using not only Alb but also PNI. PNI is calculated by Alb and total lymphocyte count, and Onodera et al. [26] reported that resection and anastomosis of the gastrointestinal tract can be safely practiced when the index is > 45. The same procedure may be dangerous when the PNI score is between 40 and 45, whereas this kind of operation may be contraindicated when it is below 40. In this study population, the proportion of cases with PNI less than 40 was extremely small (1/101; 1%); therefore, we set 45 as the cutoff value. Hence, no significant difference was observed. Nonetheless, a larger-scale study is needed in the future to confirm these results.

For postoperative factors, fever on POD3 and early first defecation after surgery were early predictive factors for AL. Once AL occurs, discharge may take time regardless of treatment. The median time until hospital discharge was 43 (range, 24–242 days) and 11 days post-surgery (range, 7–29 days) for the AL and non-AL groups, respectively. There was no significant difference in the time from the primary operation to hospital discharge between the AL group requiring re-operation and not requiring re-operation [median POD55 (range, 24–242 days) vs. POD42.5 (range, 24–51 days), *p* = 0.51). According to the analysis of postoperative factors, most AL occurs within POD3 and become a diagnosable symptomatic state after POD4. The time required from the occurrence of AL to the diagnosis may lead to the development of peritonitis, and thus, normalization of abdominal inflammation may take a long time. Therefore, in cases of fever on POD3 and early first defecation after surgery, the onset and diagnosis of AL should be monitored by fasting management and image inspection to prevent peritonitis.

In addition, it has been reported that early first defecation after surgery is a risk factor for AL [27]. Instrumental DST anastomosis within POD7 is insufficient for completion of epithelialization. Therefore, early endoluminal pressure of the first defecation is considered to be a risk factor of AL. Some studies reported that the placement of a transanal drain could prevent AL by reducing endoluminal pressure around the anastomotic site [28–32]. There are slight differences in each subject, such as the material, diameter, length of insertion, and duration of the transanal drain. A standardized procedure for placement of the transanal drain should be validated, and further investigation is required to elucidate its usefulness. With regard to reducing the endoluminal pressure around the anastomotic site, the concept of DS is similar; however, construction of DS increases patients’ discomfort and requires further surgery for stoma closure. Therefore, if the efficacy of the prevention of AL is approximately equal for both procedures, a transanal drain is superior to DS. Although there was no statistically significant difference in this study, we predict that the replacement of a transanal drain would be useful to reduce the AL rate.

Some limitations of this study have to be addressed. First, the major limitations of our study are the single-institution, retrospective design and small number of patients investigated. In fact, the AL rate in this study was slightly higher in percentage (13%). This is probably because the present study included the cases in the introduction phase of laparoscopic LAR during standardization of procedures. Furthermore, since advanced cases were not indicated for laparoscopic surgery during the introduction period, it is necessary to consider the possibility of selection bias in factors such as tumor size and TNM classification. Second, DS cases were excluded from the present study because we included only symptomatic AL. This may have also contributed to the high AL rate in this study. Moreover, a lot of DS cases have Rb lesions, which may cause selection bias. Third, preoperative chemotherapy or chemoradiotherapy cases were also excluded from this study because of our treatment policy. Among the cases of preoperative treatment, some highly advanced and difficult cases were included, which may have caused a bias in our results. These limitations should be considered when evaluating the results of the present study. It is necessary to carry out a prospective study with multiple institutions that have a unified definition of AL and standardized procedures.

## Conclusion

In conclusion, we demonstrated that in patients with AL after laparoscopic LAR with DST anastomosis, intraoperative blood loss was an indicator of difficulty in a transection and anastomosing procedure, and fever-POD3 (≥ 38 °C) and early first defecation day after surgery (< POD3) were independent early predictive factors.

Therefore, careful surgery using an appropriate technique and standardized procedures with minimal bleeding and careful postoperative management paying attention to fever and defecation may prevent the onset and severity of AL.

However, because of the retrospective nature of this study, the limited number of patients, and the multifactorial nature of AL, it is difficult to draw robust conclusions. Further studies that are multi-institutional, randomized, and controlled are required to identify risk and early predictive factors for AL.

## Data Availability

The datasets used during this study are available from the corresponding author on reasonable request.

## Abbreviations

AL: Anastomosis leakage
Alb: Albumin
BMI: Body mass index
CI: Confidence interval
CRP: C-reactive protein
DS: Diverting stoma
DST: Double-stapling technique
LAR: Low anterior resection
OR: Odds ratio
POD: Postoperative day
Ra: Upper rectal
Rb: Lower rectal
Rs: Rectosigmoid
WBC: White blood cells
